# Comparative expression analysis of Septin 14 in testes of infertile men with normal spermatogenesis and spermatogenic failure

**Published:** 2014-03

**Authors:** Maryam Shafipour, Marjan Sabbaghian, Maryam Shahhoseini, Mohammad Ali Sadighi Gilani

**Affiliations:** 1 *Department of Andrology, Reproductive Biomedicine Research Center, Royan Institute, ACECR, Tehran, Iran.*; 2 *Department of Reproductive Genetics, Reproductive Biomedicine Research Center, Royan Institute, ACECR, Tehran, Iran.*

**Keywords:** *Septin 14*, *Expression*, *Spermatogenesis*, *Male infertility*, *Azoospermia*

## Abstract

**Background:** Septins are an evolutionary conserved group of GTP-binding and filament-forming proteins that have diverse cellular roles. An increasing body of data implicates the septin family in the pathogenesis of diverse states including cancers, neurodegeneration, and male infertility.

**Objective:** The objective of the study was to evaluate the expression pattern of Septin14 in testis tissue of men with and without spermatogenic failure.

**Materials and Methods: **The samples retrieved accessible random between infertile men who underwent diagnostic testicular biopsy in Royan institute. 10 infertile men with obstructive azoospermia and normal spermatogenesis and 20 infertile men with non-obstructive azoospermia were recruited for real-time reverse transcription (RT)-PCR analysis of the testicular tissue. Total RNA was extracted with trizol reagent.

**Results: **Comparison of the mRNA level of septin14 revealed that in tissues with partial (n=10) or complete spermatogenesis (n=10), the expression of septin 14 was significantly higher than sertoli cell only tissues.

**Conclusion:** The testicular tissues of men with hypospermatogenesis, maturation arrest and sertoli cell only had lower levels of septin 14 transcripts than normal men. These data indicates that Septin 14 expression level is critical for human spermatogenesis.

## Introduction

The septins are a family of GTPases expressed in a variety of species, ranging from single-celled yeasts to humans (1-2). Septins were identified more than 35 years ago in *Saccharomyces cerevisiae* mutants that were defective in cytokinesis and cell morphology. Shortly thereafter, electron microscopy (EM) studies of budding yeast identified a series of 10-nm filaments from septins that encircle the neck between mother cells and buds (3-4). In mammals, 14 septin genes have been identified so far (5-6). They have diverse cellular roles including polarity determination, cytoskeletal reorganization, membrane dynamics, vesicle trafficking, mitosis, and exocytosis (7). 

Disruption of septin functions has been implicated in the pathology of many diseases, including neoplasia, neurodegenerative conditions, sporadic breast cancer, Parkinson and male infertility (8-11). Each septin needs to proximity to other septins to do functions. They form smaller, core complexes both in vivo and in vitro that contain, depending on the organism, two, three or four septins, each present in two copies. These core tetra-, hexa- or octameric complexes are thought to define the building blocks that are further assembled into various higher-order structures, including filaments, gauzes and rings (12-14). Often, it is seen in relation to cell elements such as tubulin & actin (15).

The first evidence that septins may contribute to human disease occurred with the discovery of SEPT5 (CDCREL-1) as a carboxy-terminus fusion partner with mixed-lineage leukemia (MLL) in an acute myeloid leukemia (AML) patient with a t (11; 22) (q23; q11.2) re-arrangement (16-18). The roles of septins in mammalian reproduction are just beginning to be revealed (19-20). Recent studies reported abundant expression of septins in testis tissues. Septins are the main components that forming annulus rings; a region of the mammalian sperm flagellum connecting the midpiece and the principal piece (21-23).

Here, we study one of the new members of septin family called septin14 that specially expressed in testis*. *SEPT14 maps to 7p11.2 in humans and includes a conserved GTPase domain and a predicted carboxy-terminus coiled-coil domain characteristic of other septins (16). Biochemical analyses revealed that C-terminal coiled-coil region of Sept14 interacts with Septin 4. In addition Sept14 is involved in neuronal migration in cerebral cortex via interaction with Sept4 (5). Peterson *et al* showed that RT-PCR expression is better for detection of Sept 14 in normal testis tissues and suggested that Sept14 can operate as a testis-specific tumor suppressor (16). In this study; for the first time we measured the expression level of septin14 in human testis tissues with normal and spermatogenic failure to reveal the dynamic role of sept14 in fertility. 

## Materials and methods

The research is an experimental study, samples were retrieved from infertile men who underwent diagnostic testicular biopsy in Royan institute from May to July 2010. We recruited 30 infertile men presenting with azoospermia confirmed by analysis of at least two different semen specimens. The diagnosis of obstructive azoospermia with normal spermatogenesis was confirmed by testicular biopsy. 10 Infertile men with obstructive azoospermia and normal spermatogenesis and 20 Infertile men with non-obstructive azoospermia that were subdivided in two groups, maturation arrest & Sertoli cell only were included. Sept14 mRNA quantification was performed in triplicate by quantitative real-time PCR (Q-PCR) on a 750 REAL-Time PCR system (Applied Bio systems), using SYBER Green master mix (Applied Bio systems), with the following primers:

F: 5' ACAAAGAAGC ATATCTCGGA 3'

R: 5' ACAGATGAAG TGAAAGTTGG 3'

The mean threshold cycle value for each complementary DNA sample was expressed as an arbitrary value relative to the standard curve after linear regression analysis. Each experiment was repeated three times. Data were normalized with β-actin values. Clinical variables are the mean±SD, and quantitative PCR results are the mean±SEM derived from the number of different experiments. 

**Statistical analysis**

Statistical analyses were performed with use of ANOVA with SPSS version 18, p<0.05 were considered significant. 

## Results

**Figure 1:**
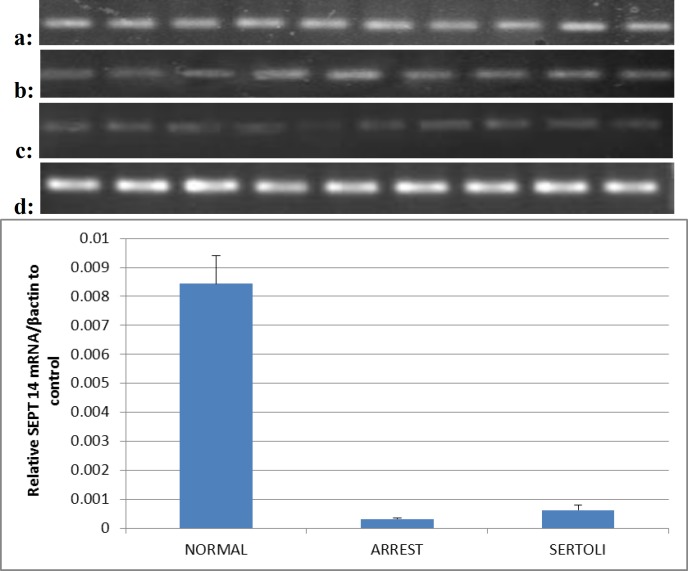
Quantitative reverse transcription PCR analysis of Sept 14 expression in human testis tissues, 10 samples in each group. (a) Expression of Sept14 in normal spermatogenesis group, (b) arrest group (c) Sertoli cell only group and (d) Expression of β-actin in human testis tissues. (mean±SD, * p<0.05)

Based on descriptive statistics, average of septin 14 placed in 3 groups of Sertoli cell only, maturation arrest and normal, in respective 0.0006±0.00016, 0.0003±0.0004, 0.0084±0.00098. As shown in Figure 1 statistically significant differences for the SEPTIN14 transcript ration were noted between Sertoli cell-only group and normal group (p<0.05). 

In addition the level of sept14 mRNA revealed that in tissues with maturation arrest (n=10), the expression of septin 14 were significantly lower than normal tissues. For Infertile men with non-obstructive azoospermia, the septin14 transcript ration was not significantly different in Sertoli cell-only infertile men and those with maturation arrest.

## Discussion

Growing evidence has suggested mammalian septins interact with diverse molecules to ensure completion of cytokinesis in somatic cells. In somatic cells, the mother-daughter cells are connected transiently by an intercellular bridge at the termination of cytokinesis, followed by abscission, or cutting of mid-body channel (24). In germ cells, the intercellular bridges of germ cells are transformed into a stable structure. The germ cell intercellular bridge is evolutionarily conserved from invertebrate to mammals (2). Disorder of structural elements of the intercellular bridge will result in sterility. The septins are main laments that filling gaps between cells, so any distribution in expression of septin may affect fertility in men (9). 

Septin 4 and septin 12 play important roles in spermatogenesis and morphology of sperm (19). In the Sept4 null mice the absence of Sept4 caused the complete loss of the annulus, suggesting that Sept4 is a critical component of this structure. The lack of a functional annulus caused a structural defect in spermatozoa resulting in failure of kinesin-dependent flagella motion. Also SEPT12 knock-out mice showed spermatogenic defects (1, 19). Septin14 is a new member of the septin family discovered by Peterson in 2007 when he was working on septin9 (16). In this study, we analyzed transcript levels of Sept 14 in testicular tissue specimens from infertile men with azoospermia. 

It was shown that the expression level of the protein is directly depending on spermatogenesis progress in testis tissue. Fig1 is shown expression of sept14 in human testis tissues with RT-PCR analysis and evaluated the relationship between these mRNA transcript levels, and stages of spermatogenesis by real-time reverse transcription-polymerase chain reaction (Q-PCR). Peterson and colleagues, in study on septin9 in human testis following yeast two-hybrid isolation, identified septin9 binding partners. They called this protein Sept14. They stated that septin14 colocalized with septin9 when co expressed (16). Tomoy *et al* in a study on transmission of nervous in members of septin family, including septin14, suggested that sept14 is involved in transmission of nervous in cerebral cortex via interaction with sept4. 

In this study, for the first time, we investigated the relationship between sept14 mRNAs and pathogenesis. We reviewed expression of septin14 in azoospermic men. Comparison of the expression levels of septin 14 revealed that the testicular tissues of men with hypo spermatogenesis, maturation arrest and Sertoli cell only had lower levels of septin 14 transcripts than normal men. In the other words, maximum level expression of septin14 occurred after spermatid stage and during maturation process.

## Conclusion

It was shown that septin 14 expression levels are critical for human spermatogenesis and decreased expression is associated with the pathogenesis of male infertility. These results indicate that the measurement of septin 14 transcript levels potentially may be useful in predicting the presence of sperm in testis. If we fail to identify spermatozoa, despite detecting high expressions of these transcripts, it may be worthwhile to repeat TESE (diagnostic testicular biopsy) until the success of sperm retrieval.
